# Lower serum triglyceride levels linked to more severe motor performance in Parkinson’s disease

**DOI:** 10.1007/s10072-022-06113-9

**Published:** 2022-05-24

**Authors:** Meimei Zhang, Huimin Chen, Genliang Liu, Xuemei Wang, Zhan Wang, Tao Feng, Yumei Zhang

**Affiliations:** 1 Department of Neurology, Beijing Tiantan Hospital, Capital Medical University, No.119 South 4th Ring West Road, Fengtai District, Beijing, 100070 China; 2 Department of Neurology, Beijing Hospital, National Center of Gerontology, Beijing, China; 3 China National Clinical Research Center for Neurological Diseases (NCRC-ND), Beijing, China; 4 Department of Rehabilitation, Beijing Tiantan Hospital, Capital Medical University, No.119 South 4th Ring West Road, Fengtai District, Beijing, 100070 China

**Keywords:** Parkinson’s disease, Motor performance, Gait, Triglyceride, Lipid

## Abstract

**Introduction:**

Emerging evidence has suggested that lipid metabolism is correlated with Parkinson’s disease (PD) onset and progression. However, the effect of lipid metabolism on motor performance in PD patients is still unknown. This study estimated the association between lipid profiles and the severity of motor performance in PD.

**Methods:**

This cross-sectional study enrolled 279 idiopathic PD patients from the Department of Neurology of Beijing Tiantan Hospital from May 2016 to August 2018. Serum triglyceride (TG), total cholesterol (TC), low-density lipoprotein cholesterol (LDL-C), high-density lipoprotein cholesterol (HDL-C), apolipoprotein A1 (Apo-A1), and apolipoprotein B (Apo-B) levels were detected in fast serum samples. Motor performance was assessed by Movement Disorder Society-Unified Parkinson’s Disease Rating Scale part III (MDS-UPDRS III) total scores and subscores in these patients. The associations of lipid profiles with motor performance were analyzed using multivariable linear regression models.

**Results:**

Compared to males, females with PD exhibited significantly higher serum TC, LDL-C, HDL-C, Apo-A1, and Apo-B levels. When accounting for covariates, lower serum TG levels were significantly associated with higher MDS-UPDRS III total scores and gait/postural instability subscores. Additionally, the univariate linear regression model showed that in males with PD, serum HDL-C or Apo-A1 levels were significantly associated with tremor subscores.

**Conclusion:**

Lower serum TG levels were associated with more severe motor performance in patients with PD and TG may be a potential predictive biomarker for motor performance in PD patients.

**Supplementary Information:**

The online version contains supplementary material available at 10.1007/s10072-022-06113-9.

## Introduction

Parkinson’s disease (PD) is the second most common neurodegenerative disease in the world and is characterized by a variety of motor and nonmotor symptoms. Cardinal motor symptoms, including resting tremor, rigidity, bradykinesia, and gait/postural instability, are usually the initial presentation and seriously affect the daily activities of patients. Action and postural tremors are also observed in PD patients and are considered variant types of resting tremor [1, 2]. In addition to the well-known α-synuclein aggregation, neuroinflammation is another pathological feature of PD [3].

Lipid metabolism is a well-established risk factor for cerebrovascular disease and cardiovascular disease and regulates postischemic inflammation [4]. However, increasing evidence indicates that lipid metabolism is correlated with PD onset and progression. In a longitudinal large-scale Israeli cohort study including statin-free individuals, a 0.3% (746 cases) PD incidence was detected after a mean 7.9-year follow-up, and they found higher levels of serum total cholesterol (TC) and low-density lipoprotein cholesterol (LDL-C) in men over time, indicating a decreased PD risk [5]. Another large cohort study in Switzerland showed that increasing levels of triglyceride (TG), TC, LDL-C, and apolipoprotein B (Apo-B) were associated with a decreased risk of PD, and the association did not differ between men and women [6].

There are few studies on the correlation between lipid metabolism and motor and nonmotor performance in PD patients and the existing studies have reported inconsistent results. A study from China showed that levels of serum TC and LDL-C were negatively correlated with Unified Parkinson’s Disease Rating Scale part III scores [7]. Lipid subfractions may be associated with nonmotor symptoms in individuals with PD. Studies reported that higher serum TG levels were associated with PD with mild cognitive impairment [8] and lower anxiety levels [9]. Another study showed that compared to males with PD, females with PD had higher serum HDL-C levels, which were potentially associated with poorer cognition performance [10]. However, Choe et al. found that no lipid fraction was significantly associated with motor and cognitive function in PD patients in a cross-sectional analysis of the Biomarkers in Parkinson’s Disease (Mark-PD) study [11].

Whether lipid metabolism factors can be utilized as PD disease severity biomarkers have not been clarified, although a study from Parkinson’s Progression Markers Initiative (PPMI) showed that lower serum apolipoprotein A1 (Apo-A1) levels were associated with earlier age of PD onset and greater motor severity in drug-naïve PD [12]. The effect of lipid metabolism on individual motor symptoms, such as tremor, rigidity, bradykinesia, and gait instability, has not been investigated. In addition, previous studies considered limited lipid measurements, mostly TC alone or TC with LDL-C and high-density lipoprotein cholesterol (HDL-C), but not the effects of whole lipid profiles.

Therefore, the aim of our study was to estimate the association of relatively complete lipid profiles with the severity of global motor performance and four carinal motor symptoms in PD patients.

## Methods

### Participants

This cross-sectional study enrolled 279 idiopathic PD patients from the Department of Neurology of Beijing Tiantan Hospital, Capital Medical University, from May 2016 to August 2018. Patients who met the clinically definite PD based on 2015 Movement Disorder Society (MDS) Clinical Diagnosis Criteria were included [13]. The exclusion criteria included (1) an uncertain PD diagnosis or suspicion of atypical parkinsonism (multiple system atrophy, corticobasal ganglionic degeneration, or progressive supranuclear palsy) or second parkinsonism syndrome (vascular, drug-induced, toxin-induced, or postinfectious parkinsonism); (2) a history of cerebral infarction or coronary atherosclerotic heart disease; or (3) a history of moderate-to-severe head trauma, hydrocephalus, brain surgery, or brain tumor. This study was approved by the Ethics Committee of the Beijing Tiantan Hospital and was performed in accordance with the Declaration of Helsinki. Informed consent was obtained from either the participant or their closest relative.

### Clinical assessment of participants

The demographic data (age at admission, sex, weight and height, and disease duration) of all the enrolled PD patients were collected. The use of statin or other lipid-lowering medicines was also recorded. The severity of PD was assessed by Hoehn–Yahr (H-Y) staging and the Movement Disorder Society-Unified Parkinson’s Disease Rating Scale part III (MDS-UPDRS III) total score. The subscores for tremor (items 3.15, 3.16, 3.17, and 3.18), rigidity (items 3.3), bradykinesia (items 3.4, 3.5, 3.6, 3.7, 3.8, 3.9, and 3.14), and gait/postural instability (items 3.10, 3.11, and 3.12) were obtained from the MDS-UPDRS III. Cognition was assessed by the Montreal Cognitive Assessment (MoCA). Mood was assessed by the Hamilton Anxiety Rating Scale (HAMA) and Hamilton Depression Rating Scale (HAMD). Motor assessment was performed in an off-medication state, which was defined after 12 h of overnight withdrawal from antiparkinsonian medications; however, cognition and mood assessments were performed in on/off-medication state (the on-medication state was defined after 1.5–2 h from antiparkinsonian medications intaking). The levodopa equivalent daily dosage (LEED) was calculated as levodopa dose + levodopa dose × 1/3 if on entacapone + piribedil (mg) + pramipexole (mg) × 100 + selegiline (mg) × 10 + amantadine (mg) + controlled-release levodopa (mg) × 0.75 [14]. All individuals with PD were assessed by experienced neurologists.

### Laboratory assessment

Fast serum samples were obtained in the morning and serum levels of lipid profiles, including TC, LDH-C, HDL-C, TG, Apo-A1, and Apo-B, were measured in the clinical laboratory of the Beijing Tiantan Hospital. The levels of serum TC, TG, LDL-C, and HDL-C were determined using enzymological methods while the levels of Apo-A1 and Apo-B were determined using immunoturbidimetric assays. Serum uric acid and homocysteine were also detected.

### Statistical analysis

All statistical analyses were performed in SPSS 24.0. The Kolmogorov–Smirnov test was used to check the distribution of the data. Continuous variables were expressed as means and standard deviation (SD) or median and interquartile range (IQR) accordingly to normality of the data, and categorical variables were reported as numbers and percentages. Student’s *t*-test and Mann–Whitney *U* test were used for comparison of normally and nonnormally distributed data between males and females, respectively. Chi-square test was used to evaluate differences in categorical variables.

We assessed the association between lipid profiles and motor performance in PD using univariate and multivariable linear regression models. We used motor performance (MDS-UPDRS III total scores, tremor subscores, rigid subscores, bradykinesia subscores, or gait/postural instability subscores) as the dependent variable, and the serum lipid levels (TG, TC, LDL, HDL, Apo-A1, or Apo-B) as the independent variables. Data with missing outcomes were removed from the analysis. Two multivariable linear regression models were conducted. Model 1 accounted for demographic and clinical variables, including age, education, body mass index (BMI), age at onset, disease duration, MoCA score, HAMA score, HAMD score, LEED, and use of lipid-lowering medication. Model 2 additionally accounted for other biomarkers reported to affect motor performance in PD patients, such as uric acid and homocysteine [15]. Unstandardized (*B*) or standardized coefficients (*β*) and their 95% confidence intervals were calculated. The level of significance was *p* < 0.05 (two-sided).

## Results

### Demographic and clinical information

The demographic and clinical characteristics of the participants were summarized in Table 1. A total of 279 PD patients with a median age at assessment of 64.00 years old, median disease duration of 6.00 years, and median age of PD onset of 57.00 years were enrolled in our study, and 56.99% were males. There were no significant sex-specific differences in age, disease duration, age at onset, BMI, use of lipid-lowering medicines, H-Y staging, MDS-UPDRS III total scores, and LEED (Table 1). Males had more education years, higher MoCA scores, and lower HAMA and HAMD scores than females (Table 1). Subsequent analyses of the association between lipid profiles and motor performance therefore included MoCA and HAMA scores as covariates, as well as other demographic and clinical variables.

**Table 1:** Sex differences in demographic and clinical features in PD

	Total (*n* = 279)	Males (*n* = 159)	Females (*n* = 120)	*p*
Age (years)	64.00 (58.00, 69.00)	64.00 (57.00, 70.00)	64.50 (58.00, 68.75)	0.641
BMI (kg/m^2^)	24.22 (22.04, 26.50)	24.34 (22.84, 26.57)	23.44 (21.48, 26.32)	0.056
Lipid-lowering medicine, *n* (%)	14 (5.02%)	7 (4.40%)	7 (5.83%)	0.593
Education (years)	10.00 (8.00, 12.00)	12.00 (9.00, 15.00)	9.00 (6.00, 12.00)	** < 0.001**
Age at onset (years)	57.00 (50.00, 62.00)	55.00 (50.00, 62.00)	57.50 (50.00, 62.00)	0.670
Disease duration (years)	6.00 (4.00, 10.00)	6.00 (4.00, 10.00)	7.00 (4.00, 10.00)	0.221
H-Y stage	3.00 (2.00, 4.00)	3.00 (2.00, 3.00)	3.00 (2.00, 4.00)	0.084
MDS-UPDRS III total score	39.00 (29.00, 52.00)	38.00 (29.00, 52.00)	41.00 (28.00, 52.75)	0.505
Tremor subscores	6.00 (3.00, 10.50)	6.00 (2.00, 9.50)	6.50 (3.00, 11.00)	0.131
Rigid subscores	8.00 (4.00, 10.00)	8.00 (5.00, 10.75)	8.00 (4.00, 10.00)	0.442
Bradykinesia subscores	23.00 (14.00, 31.25)	22.00 (15.00, 30.50)	24.00 (13.00, 32.00)	0.750
Gait/postural instability subscores	4.00 (2.00, 6.00)	3.00 (1.25, 5.00)	4.00 (2.00, 7.00)	**0.002**
LEED (mg/day)	475.00 (300.00, 675.00)	450.00 (300.00, 650.00)	500.00 (300.00, 739.00)	0.348
MoCA score	23.00 (17.00, 26.00)	24.00 (20.00, 26.00)	22.00 (15.00, 25.00)	**0.004**
HAMA score	13.00 (8.00, 19.00)	11.00 (6.00, 11.50)	16.00 (9.50, 22.00)	** < 0.001**
HAMD score	12.00 (6.00, 18.00)	10.00 (5.00, 16.00)	15.00 (9.00, 22.00)	** < 0.001**

Nonnormal distributed continuous variables were expressed as median (IQR) and categorical variables expressed as number (percentage %). Statistically significant results are shown in bold

Abbreviations: *BMI*, body mass index; *H-Y staging*, Hoehn–Yahr staging; *MDS-UPDRS III*, Movement Disorder Society-Unified Parkinson’s Disease Rating Scale part III; *LEED*, levodopa equivalent daily dosage; *MoCA*, Montreal Cognitive Assessment; *HAMA*, Hamilton Anxiety Rating Scale; *HAMD*, Hamilton Depression Rating Scale

### Sex-based differences within lipid profiles in PD

To explore the association of lipids with motor performance considering sex differences in lipid profiles, a total of 159 males and 120 females with PD were included and lipids were measured. The mean level of TG was 1.21 mmol/L, TC was 4.10 mmol/L, LDL-C was 2.44 mmol/L, HDL-C was 1.23 mmol/L, Apo-A1 was 1.35 g/L, and Apo-B was 0.86 g/L.

**Fig. 1:**
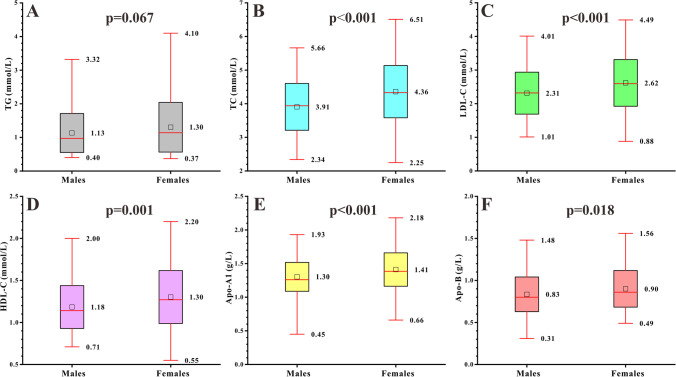
Sex differences in lipid profiles in PD. Boxplots (**A**–**F**) show the differences between males and females with PD, including TG, TC, LDL-C, HDL-C, Apo-A1, and Apo-B. Abbreviations: TG, triglyceride; TC, total cholesterol; LDL-C, low-density lipoprotein cholesterol; HDL-C, high-density lipoprotein cholesterol; Apo-A1, apolipoprotein A1; Apo-B, apolipoprotein B

We compared lipid profiles between males and females with PD, as sex-specific differences in lipid profiles are often reported in healthy populations [16, 17] and a study showed higher TC and LDL-C levels associated with lower PD risk in males [5]. We observed significant differences between male and female participants in serum levels of TC (*p* < 0.001), LDL-C (*p* < 0.001), HDL-C (*p* = 0.001), Apo-A1 (*p* < 0.001), and Apo-B (*p* = 0.018) (Fig. 1). Such a difference was not found in serum TG levels (*p* > 0.05) (Fig. 1). Therefore, in order to accurately explore the effects of lipid profiles on motor performance in patients with PD, the sex-based analyses were considered necessary and were employed for further statistical analyses concerning TC, LDL-C, HDL-C, Apo-A1, and Apo-B. However, analysis of TG levels was conducted in males and females together.

Considering the missing information for MDS-UPDRS III subitem scores of 12 participants, we finally enrolled 267 PD participants in the following linear regression analysis.

### Motor performance and TG levels in PD

The univariate linear regression model showed that lower serum TG levels were significantly associated with higher MDS-UPDRS III total scores, rigidity subscores, bradykinesia subscores, and gait/postural instability subscores in PD patients (*p* < 0.05) (Table 2). The multivariable linear regression models (models 1 and 2) showed that lower serum TG levels were significantly associated with higher MDS-UPDRS III total scores and gait/postural instability subscores (*p* < 0.05) (Table 2); no significant association was shown between TG levels and rigid subscores and bradykinesia subscores in the two models. Serum TG levels were not significantly associated with tremor subscores in either the univariate or multivariable linear regression model (*p* > 0.05) (Table 2).

**Table 2:** Motor performance based on TG levels, total study population

	Univariate		Model 1		Model 2	
	Unstandardized *B* coefficient (95% CI)	*p*	Standardized *β* coefficient (95% CI)	*p*	Standardized *β* coefficient (95% CI)	*p*
MDS-UPDRS III total scores	− 5.249 (− 8.527, − 1.977)	**0.002**	− 0.189 (− 10.106, − 1.424)	**0.010**	− 0.172 (− 9.732, − 0.911)	**0.018**
Tremor subscores	− 0.543 (− 1.811, 0.726)	0.400	− 0.034 (− 1.826, 1.202)	0.686	− 0.034 (− 1.850, 1.229)	0.691
Rigid subscores	− 0.955 (− 1.866, − 0.044)	**0.040**	− 0.110 (− 1.885, 0.376)	0.189	0.118 (− 1.958, 0.339)	0.166
Bradykinesia subscores	− 3.145 (− 5.912, − 0.379)	**0.026**	− 0.144 (− 6.262, 0.311)	0.076	− 0.141 (− 6.254, 0.418)	0.086
Gait/postural instability subscores	− 0.898 (− 1.591, − 0.206)	**0.011**	− 0.195 (− 1.956, − 0.168)	**0.020**	− 0.196 (− 1.975, − 0.156)	**0.022**

Multivariable linear regression model 1 was adjusted for age, education, BMI, age at onset, disease duration, MoCA scores, HAMA scores, HAMD scores, LEED, and use of lipid-lowering medication; model 2 was further adjusted for uric acid and homocysteine levels. Statistically significant results are shown in bold

Abbreviations: *TG*, triglyceride; *CI*, confidence interval; *MDS-UPDRS III*, Movement Disorder Society-Unified Parkinson’s Disease Rating Scale part III; *BMI*, body mass index; *MoCA*, Montreal Cognitive Assessment; *HAMA*, Hamilton Anxiety Rating Scale; *HAMD*, Hamilton Depression Rating Scale; *LEED*, levodopa equivalent daily dosage

### Motor performance and TC levels in PD

The univariate and multivariable linear regression models (models 1 and 2) showed that serum TC levels were not significantly associated with motor performance in males or females with PD (*p* > 0.05) (Supplementary Table 1).

### Motor performance and LDL-C levels in PD

The univariate and multivariable linear regression models (models 1 and 2) showed that serum LDL-C levels were not significantly associated with motor performance in males or females with PD (*p* > 0.05) (Supplementary Table 2).

### Motor performance and HDL-C levels in PD

The univariate linear regression model showed that higher HDL-C levels were significantly associated with higher tremor subscores in males (*p* < 0.05), while only tendencies toward significant associations (*P* < 0.1) were found in model 1 and model 2 (Table 3). There were no significant associations between HDL-C levels and MDS-UPDRS III total scores, rigidity subscores, bradykinesia subscores, and gait/postural instability subscores in males (*p* > 0.05) (Table 3). The univariate and multivariable linear regression models (models 1 and 2) showed that serum HDL-C levels were not significantly associated with motor performance in females with PD (*p* > 0.05) (Table 3).

**Table 3:** Motor performance based on HDL-C levels by sex

	Univariate		Model 1		Model 2	
	Unstandardized *B* coefficient (95% CI)	*p*	Standardized *β* coefficient (95% CI)	*p*	Standardized *β* coefficient (95% CI)	*p*
Males						
MDS-UPDRS III total scores	7.189 (− 3.874, 18.253)	0.201	0.079 (− 10.280, 21.977)	0.473	0.099 (− 8.704, 23.359)	0.366
Tremor subscores	5.450 (1.000, 9.899)	**0.017**	0.223 (− 0.296, 11.175)	0.063	0.227 (− 0.294, 11.345)	0.062
Rigid subscores	− 0.142 (− 3.229, 2.945)	0.928	− 0.055 (− 5.342, 3.322)	0.643	− 0.066 (− 5.583, 3.128)	0.576
Bradykinesia subscores	0.802 (− 8.578, 10.138)	0.866	− 0.017 (− 13.771, 11.815)	0.879	− 0.012 (− 13.542, 12.223)	0.919
Gait/postural instability subscores	2.063 (− 0.110, 4.236)	0.063	0.204 (− 0.382, 6.124)	0.083	0.189 (− 0.298, 6.188)	0.074
Females						
MDS-UPDRS III total scores	4.830 (− 6.258, 15.919)	0.390	0.077 (− 8.151, 17.443)	0.472	0.048 (− 10.250, 16.005)	0.663
Tremor subscores	0.626 (− 3.042, 4.294)	0.735	0.011 (− 4.448, 4.841)	0.933	0.013 (− 4.574, 5.045)	0.922
Rigidity subscores	0.471 (− 2.378, 3.319)	0.743	0.037 (− 2.961, 3.979)	0.770	0.017 (− 3.337, 3.809)	0.895
Bradykinesia subscores	− 0.135 (− 8.842, 8.571)	0.975	0.025 (− 8.776, 10.841)	0.834	0.004 (− 9.936, 10.255)	0.975
Gait/postural instability subscores	1.121 (− 1.109, 3.351)	0.321	0.091 (− 1.845, 3.785)	0.493	0.034 (− 2.411, 3.129)	0.796

Multivariable linear regression model 1 was adjusted for age, education, BMI, age at onset, disease duration, MoCA scores, HAMA scores, HAMD scores, LEED, and use of lipid-lowering medication; model 2 was further adjusted for uric acid and homocysteine levels. Statistically significant results are shown in bold

Abbreviations: *HDL-C*, high-density lipoprotein cholesterol; *CI*, confidence interval; *MDS-UPDRS III*, Movement Disorder Society-Unified Parkinson’s Disease Rating Scale part III; *BMI*, body mass index; *MoCA*, Montreal Cognitive Assessment; *HAMA*, Hamilton Anxiety Rating Scale; *HAMD*, Hamilton Depression Rating Scale; *LEED*, levodopa equivalent daily dosage

### Motor performance and Apo-A1 levels in PD

The univariate linear regression model showed that higher Apo-A1 levels were significantly associated with higher tremor subscores (*p* < 0.05), while only tendencies toward significant associations (*p* < 0.1) were found in model 1 and model 2 (Table 4). There were no significant association between Apo-A1 levels and MDS-UPDRS III total scores, rigidity subscores, bradykinesia subscores, and gait/postural instability subscores in males (*p* > 0.05) (Table 4). The univariate and multivariable linear regression models (models 1 and 2) showed that serum Apo-A1 levels were not significantly associated with motor performance in females with PD (*p* > 0.05) (Table 4).

**Table 4:** Motor performance based on Apo-A1 levels by sex

	Univariate		Model 1		Model 2	
	Unstandardized *B* coefficient (95% CI)	*p*	Standardized *β* coefficient (95% CI)	*p*	Standardized *β* coefficient (95% CI)	*p*
Males						
MDS-UPDRS III total scores	4.219 (− 8.952, 17.391)	0.528	0.006 (− 16.073, 16.992)	0.956	0.009 (− 15.720, 17.104)	0.933
Tremor subscores	6.748 (1.645, 11.851)	**0.010**	0.203 (− 0.488, 10.654)	0.073	0.201 (− 0.599, 10.679)	0.079
Rigid subscores	− 0.622 (− 4.177, 2.933)	0.730	− 0.050 (− 5.162, 3.240)	0.650	− 0.054 (− 5.239, 3.183)	0.628
Bradykinesia subscores	− 2.440 (− 13.241, 8.360)	0.655	− 0.057 (− 15.672, 9.094)	0.598	− 0.060 (− 15.888, 8.956)	0.580
Gait/postural instability subscores	0.894 (− 1.641, 3.429)	0.486	0.047 (− 2.534, 3.900)	0.674	0.042 (− 2.590, 3.814)	0.704
Females						
MDS-UPDRS III total scores	− 0.215 (− 14.328, 13.897)	0.976	− 0.007 (− 16.317, 15.210)	0.944	− 0.027 (− 17.959, 13.876)	0.799
Tremor subscores	0.518 (− 4.142, 5.178)	0.826	0.228 (− 4.975, 6.254)	0.752	0.032 (− 5.064, 6.514)	0.803
Rigidity subscores	0.055 (− 3.562, 3.673)	0.976	0.013 (− 3.982, 4.417)	0.918	− 0.003 (− 4.355, 4.253)	0.981
Bradykinesia subscores	− 5.043 (− 16.044, 5.958)	0.365	− 0.057 (− 14.776, 8.909)	0.622	− 0.077 (− 16.080, 8.143)	0.514
Gait/postural instability subscores	− 0.198 (− 3.043, 2.648)	0.891	− 0.056 (− 1.471, 2.654)	0.658	− 0.111 (− 4.809, 1.819)	0.370

Multivariable linear regression model 1 was adjusted for age, education, BMI, age at onset, disease duration, MoCA scores, HAMA scores, HAMD scores, LEED, and use of lipid-lowering medication; model 2 was further adjusted for uric acid and homocysteine levels. Statistically significant results are shown in bold

Abbreviations: *Apo-A1*, apolipoprotein A1; *CI*, confidence interval; *MDS-UPDRS III*, Movement Disorder Society-Unified Parkinson’s Disease Rating Scale part III; *BMI*, body mass index; *MoCA*, Montreal Cognitive Assessment; *HAMA*, Hamilton Anxiety Rating Scale; *HAMD*, Hamilton Depression Rating Scale; *LEED*, levodopa equivalent daily dosage

### Motor performance and Apo-B levels in PD

The univariate linear regression model showed no significant association between Apo-B levels and motor performance in PD patients (*p* > 0.05), while the multivariable linear regression models (models 1 and 2) showed that Apo-B levels were significantly associated with gait/postural instability subscores in males with PD (*p* < 0.05) (Table 5).

**Table 5:** Motor performance based on Apo-B levels by sex

	Univariate		Model 1		Model 2	
	Unstandardized *B* coefficient (95% CI)	*p*	Standardized *β* coefficient (95% CI)	*p*	Standardized *β* coefficient (95% CI)	*p*
Males						
MDS-UPDRS III total scores	− 11.001 (− 24.634, 2.631)	0.113	− 0.170 (− 35.462, 3.649)	0.110	− 0.147 (− 33.604, 6.079)	0.172
Tremor subscores	0.815 (− 4.821, 6.452)	0.775	0.129 (− 2.997, 10.588)	0.269	0.135 (− 3.013, 10.926)	0.262
Rigid subscores	− 0.252 (− 4.120, 3.617)	0.898	0.027 (− 4.444, 5.671)	0.810	0.005 (− 5.037, 5.256)	0.966
Bradykinesia subscores	− 6.648 (− 18.335, 5.039)	0.262	− 0.108 (− 22.156, 7.487)	0.327	− 0.103 (− 22.112, 8.085)	0.357
Gait/postural instability subscores	− 1.529 (− 4.278, 1.221)	0.273	− 0.225 (− 7.581, − 0.041)	**0.048**	− 0.229 (− 7.686, − 0.085)	**0.045**
Females						
MDS-UPDRS III total scores	− 5.406 (− 21.457, 10.646)	0.506	0.046 (− 13.981, 21.656)	0.669	0.078 (− 11.564, 24.748)	0.471
Tremor subscores	− 0.218 (− 5.622, 5.186)	0.936	0.038 (− 5.765, 7.731)	0.772	0.042 (− 5.829, 8.052)	0.750
Rigidity subscores	− 1.347 (− 5.539, 2.844)	0.525	0.173 (− 1.517, 8.420)	0.170	0.194 (− 1.181, 8.935)	0.130
Bradykinesia subscores	− 8.985 (− 21.679, 3.710)	0.163	0.110 (− 7.616, 20.712)	0.359	0.125 (− 7.035, 21.860)	0.309
Gait/postural instability subscores	− 2.241 (− 5.513, 1.031)	0.177	− 0.018 (− 4.387, 3.830)	0.893	0.005 (− 3.932, 4.074)	0.972

Multivariable linear regression model 1 was adjusted for age, education, BMI, age at onset, disease duration, MoCA scores, HAMA scores, HAMD scores, LEED, and use of lipid-lowering medication; model 2 was further adjusted for uric acid and homocysteine levels. Statistically significant results are shown in bold

Abbreviations: *Apo-B*, apolipoprotein B; *CI*, confidence interval; *MDS-UPDRS III*, Movement Disorder Society-Unified Parkinson’s Disease Rating Scale part III; *BMI*, body mass index; *MoCA*, Montreal Cognitive Assessment; *HAMA*, Hamilton Anxiety Rating Scale; *HAMD*, Hamilton Depression Rating Scale; *LEED*, levodopa equivalent daily dosage

## Discussion

The present study investigated the association between lipid profiles and motor performance in patients with PD. The main result revealed that serum TG levels had a negative effect on PD motor performance independent of age, education, BMI, age at onset, disease duration, MoCA score, HAMA score, HAMD score, LEED, use of lipid-lowering medication, uric acid, and homocysteine levels. Lower serum TG levels were associated with more severe motor performance, including not only global motor performance but also gait or postural instability symptoms in patients with PD. In addition, higher serum HDL-C and Apo-A1 levels were associated with more severe tremor performance in males with PD. Lower Apo-B levels were associated with more severe gait or postural instability symptoms in males with PD.

Previous prospective studies found that higher serum TG levels were associated with lower future PD risk [6, 18] and case–control studies showed that TG levels were lower in patients with PD [19, 20]. Our findings revealed that higher TG levels were linked to better motor performance in patients with PD, which was in line with previous results that suggested that TG may play a protective role in PD.

Although the mechanisms by which TG exerts protection against PD development and motor performance are not fully understood, the biosynthesis and function of lipids make it biologically plausible. Lipids are major components of the nerve cell membranes and play important physiological roles in the brain, such as aiding in neural communication, neurogenesis, synaptic transmission, signal transduction, membrane compartmentalization, and regulation of gene expression [21, 22]. Furthermore, TG can be converted into free acids and ketones, which are highly effective energy sources for mitochondria in neurons, and TG has a protective effect on the mitochondrial function by altering reactive oxygen species release [23].

Another theory about the inverse association between TG levels and motor performance in PD is that TG reinforce dopamine-mediated behaviors. An animal study showed that increased serum TG levels after the intake of high-calorie food which entered brain served as direct positive reinforcement in the reward system, which is mediated by dopamine neurons and dopamine receptors [24].

However, there have been controversial results regarding the association between serum TG levels and PD. Huang and colleagues found that higher serum TG levels were associated with mild cognitive impairment in PD patients in a subgroup analysis [8]. The explanation was that serum TG led to a transient increase of blood–brain barrier permeability in a rat model [25]. Increased serum TG levels were associated with future increased cerebral β-amyloid and tau pathology, too [26].

Overall, the controversial results regarding the association between serum TG levels and PD warrant further exploration.

In contrast to the strong association between TG levels and motor performance in PD in the current study, associations between motor performance and TC, LDL-C, HDL-C, Apo-A1, or Apo-B levels were less robust. While higher HDL-C or Apo-A1 levels were associated with more severe tremor symptoms and lower Apo-B levels were associated with more severe gait or postural instability symptoms in males with PD, no significant associations were found for other motor symptoms in PD.

Such sex-specific differences should be considered, although the potential mechanism is largely unknown. The literatures have provided some explanations, including sex differences in cholesterol transportation and age-related cholesterol level changes [27, 28], genetically mediated lipid metabolism, steroid hormone synthesis [29], and the interaction between lipids and α-synuclein [30, 31]. What is important and cannot be ignored is the neuroprotective effects of sex hormones in females [32].

Our findings were specific to males and may be linked to male sex hormones, which do not have a neuroprotective role in diseases. Additionally, increasing evidence indicates that low-grade, chronic inflammation occurs in people with PD, and inflammation plays an important role in the genesis and pathophysiology of PD [33, 34]. It has been reported that HDL-C may become dysfunctional in circumstances of elevated inflammation, resulting the loss of anti-inflammatory and cardiovascular protective properties [35]. Apo-A1, as the main apolipoprotein of HDL-C, may have a similar effect as HDL-C on PD.

Our study also found that females have higher serum TC, LDL-C, and HDL-C levels than males in PD, which were in line with previous studies [10, 36]. Furthermore, we compared the serum Apo-A1 and Apo-B levels between sexes in PD for the first time, and females showed higher Apo-A1 and Apo-B levels in PD. Such sex differences have been shown in healthy populations, especially in males and postmenopausal females [17, 37]. Median age of females in our study was 64.50 years old, which was a postmenopausal age in Chinese women. Furthermore, the differences of serum lipid levels in sex may provide a more rational sex-based lipid management in clinic.

The study has several strengths. First, to the best of our knowledge, this is the first study to investigate the correlation between lipid profiles and various motor performance in PD patients, and reveal a novel association between serum TG levels and motor performance in patients with PD. Second, we considered the sex differences in lipid profiles to further clarify the effect of lipid profiles on motor performance in patients with PD. Third, we constructed several regression models to understand the effect of lipid profiles on motor performance in PD.

A number of limitations in the current study must be acknowledged. First, the present study was cross-sectional in nature, and the association between changes in lipid profiles levels and motor performance in PD over time was not assessed. Future longitudinal studies and larger samples are needed. Second, genetic confounding was not considered in our study, and a previous study indicated shared genetic risk between lipids and PD [38]. Third, data on other vascular risk factors, such as diet, physical exercise, smoking, alcohol consumption, menopausal status in females, and some inflammation biomarkers, were not available for analysis. Fourth, the diagnosis of PD in this study was based on clinical diagnostic criteria rather than pathology, which may affect the diagnostic accuracy.

## Conclusion

In conclusion, the novel findings of the current cross-sectional study that lower serum TG levels were linked to more severe motor performance in patients with PD provide further support for the protective role of serum TG levels in PD. Furthermore, sex-specific effects of HDL-C, Apo-A1, and Apo-B levels on tremor or gait and posture performance suggest different mechanisms underlying motor performance in males and females with PD. Lipids, as the modifiable factors, should be considered in PD patients, and further studies need to explore the underlying mechanisms to maximize the effect of lipid profiles control on PD.

## Supplementary Information

Below is the link to the electronic supplementary material.

Supplementary file1 (DOCX 19 KB)

## Data Availability

The raw data supporting the conclusions of this article will be made available by the authors, without undue reservation.
