# Supplementary material for: Lower serum triglyceride levels linked to more severe motor performance in Parkinson’s disease
Source: Neurol Sci. 2022 May 24;43(9):5343–53. doi: 10.1007/s10072-022-06113-9 (PMC9385747; doi:10.1007/s10072-022-06113-9)
Supplement: Supplementary file 1 — Supplementary file1 (DOCX 19 KB) [file 10072_2022_6113_MOESM1_ESM.docx]

**Supplementary Table 1** Motor performance based on TC levels by sex

|  | Univariate | |  | Model 1 | |  | Model 2 | |
| --- | --- | --- | --- | --- | --- | --- | --- | --- |
|  | Unstandardized B coefficient (95% CI) | p |  | Standardized β coefficient (95% CI) | p |  | Standardized β coefficient (95% CI) | p |
| **Males** |  |  |  |  |  |  |  |  |
| MDS-UPDRS III total scores | -0.442(-4.494, 3.649) | 0.838 |  | -0.033(-6.723, 4.945) | 0.763 |  | 0.014(-5.622, 6.381) | 0.900 |
| Tremor subscores | 1.027(-0.557, 2.611) | 0.202 |  | 0.200(-0.333, 3.614) | 0.102 |  | 0.213(-0.308, 3.803) | 0.094 |
| Rigid subscores | -0.023(-1.117, 1.072) | 0.967 |  | 0.055(-1.136, 1.829) | 0.643 |  | 0.021(-1.403, 1.665) | 0.865 |
| Bradykinesia subscores | -0.511(-3.836, 2.813) | 0.761 |  | 0.002(-4.341, 4.416) | 0.987 |  | 0.011(-4.325, 4.732) | 0.929 |
| Gait/postural instability subscores | 0.038(-0.744, 0.819) | 0.924 |  | -0.090(-1.559, 0.706) | 0.456 |  | -0.097(-1.621, 0.700) | 0.432 |
| **Females** |  |  |  |  |  |  |  |  |
| MDS-UPDRS III total scores | 0.254(-4.253, 4.761) | 0.911 |  | 0.108(-2.497, 7.515) | 0.321 |  | 0.108(-2.514, 7.503) | 0.324 |
| Tremor subscores | 0.835(-0.712, 2.382) | 0.287 |  | 0.102(-1.148, 2.667) | 0.429 |  | 0.112(-1.133, 2.802) | 0.399 |
| Rigidity subscores | -0.659(-1.861, 0.544) | 0.280 |  | 0.001(-1.431, 1.437) | 0.997 |  | -0.008(-1.514, 1.428) | 0.953 |
| Bradykinesia subscores | -2.340(-6.005, 1.324) | 0.208 |  | 0.051(-3.180, 4.912) | 0.670 |  | 0.037(-3.525, 4.779) | 0.763 |
| Gait/postural instability subscores | 0.181(-0.770, 1.133) | 0.706 |  | 0.105(-0.696, 1.626) | 0.426 |  | 0.055(-0.897, 1.381) | 0.672 |

Multivariable linear regression Model 1 was adjusted for age, education, BMI, age at onset, disease duration, MoCA scores, HAMA scores, HAMD scores, LEED, and use of lipid-lowering medication, Model 2 was further adjusted for uric acid and homocysteine levels.

Abbreviations: TC, total cholesterol; CI, confidence interval; MDS-UPDRS III, Movement Disorder Society-Unified Parkinson’s Disease Rating Scale part III; BMI, body mass index; MoCA, Montreal Cognitive Assessment; HAMA, Hamilton Anxiety Rating Scale; HAMD, Hamilton Depression Rating Scale; LEED, levodopa equivalent daily dosage.

**Supplementary Table 2** Motor performance based on LDL-C levels by sex

|  | Univariate | |  | Model 1 | |  | Model 2 | |
| --- | --- | --- | --- | --- | --- | --- | --- | --- |
|  | Unstandardized B coefficient (95% CI) | p |  | Standardized β coefficient (95% CI) | p |  | Standardized β coefficient (95% CI) | p |
| **Males** |  |  |  |  |  |  |  |  |
| MDS-UPDRS III total scores | -1.204(-5.738, 3.330) | 0.601 |  | -0.035(-7.923, 5.799) | 0.759 |  | 0.017(-6.566, 7.598) | 0.885 |
| Tremor subscores | 0.513(-1.272, 2.298) | 0.570 |  | 0.157(-0.842, 3.743) | 0.211 |  | 0.168(-0.845, 3.961) | 0.200 |
| Rigid subscores | 0.061(-1.169, 1.290) | 0.922 |  | 0.110(-0.931, 2.473) | 0.370 |  | 0.075(-1.248, 2.302) | 0.556 |
| Bradykinesia subscores | -0.670(-4.405, 3.065) | 0.723 |  | 0.024(-4.535, 5.559) | 0.840 |  | 0.034(-4.512, 5.984) | 0.781 |
| Gait/postural instability subscores | -0.152(-1.030, 0.726) | 0.733 |  | -0.147(-2.081, 0.515) | 0.233 |  | -0.161(-2.195, 0.478) | 0.204 |
| **Females** |  |  |  |  |  |  |  |  |
| MDS-UPDRS III total scores | -0.928(-5.974, 4.118) | 0.716 |  | 0.056(-4.261, 7.245) | 0.607 |  | 0.064(-4.066, 7.476) | 0.557 |
| Tremor subscores | 0.549(-1.154, 2.252) | 0.523 |  | 0.014(-2.060, 2.290) | 0.916 |  | 0.025(-2.056, 2.477) | 0.853 |
| Rigidity subscores | -0.771(-2.085, 0.543) | 0.247 |  | -0.009(-1.686, 1.566) | 0.942 |  | -0.013(-1.771, 1.598) | 0.919 |
| Bradykinesia subscores | -2.568(-6.578, 1.441) | 0.206 |  | 0.046(-3.706, 5.472) | 0.701 |  | 0.033(-4.122, 5.390) | 0.790 |
| Gait/postural instability subscores | -0.150(-1.191, 0.892) | 0.776 |  | 0.030(-1.172, 1.474) | 0.821 |  | -0.028(-1.448, 1.165) | 0.829 |

Multivariable linear regression Model 1 was adjusted for age, education, BMI, age at onset, disease duration, MoCA scores, HAMA scores, HAMD scores, LEED, and use of lipid-lowering medication, Model 2 was further adjusted for uric acid and homocysteine levels.

Abbreviations: LDL-C, low-density lipoprotein cholesterol; CI, confidence interval; MDS-UPDRS III, Movement Disorder Society-Unified Parkinson’s Disease Rating Scale part III; BMI, body mass index; MoCA, Montreal Cognitive Assessment; HAMA, Hamilton Anxiety Rating Scale; HAMD, Hamilton Depression Rating Scale; LEED, levodopa equivalent daily dosage.
